# Health-oriented participatory leadership in European nursing homes: a qualitative cross-country study of support for employees and teams

**DOI:** 10.1186/s12913-026-14208-8

**Published:** 2026-02-21

**Authors:** Kerstin Hämel, Caspar Lückenbach, Marcus Heumann, Thomas Gerlinger, Susanne Kümpers

**Affiliations:** 1 Department of Nursing Science, Faculty of Social Sciences, University of Vienna, Alser Strasse 23/12, Vienna, 1080 Austria; 2 Department of Health Systems, Health Policy and Health Sociology, School of Public Health, Bielefeld University, Universitätsstrasse 25, 33615 Bielefeld, Germany; 3 Department of Health Services Research and Nursing Science, School of Public Health, Bielefeld University, Universitätsstrasse 25, 33615 Bielefeld, Germany; 4 Department of Health Sciences, Fulda University of Applied Sciences, Leipziger Straße 123, 36037 Fulda, Germany

**Keywords:** Long-term care, Employee health, Work engagement, Qualitative research, Austria, The Netherlands, Sweden

## Abstract

**Background:**

In light of labour shortages, complex care needs and tough working conditions, creating health-promoting working environments in nursing homes is a challenge. Participation is a core principle of health promotion, and is highly relevant for workplace health management. In this study we focused on how management can promote workplace health with and for their employees through participation and what opportunities and challenges are associated with such approaches.

**Methods:**

For this qualitative cross-country study we conducted semi-structured interviews (*n* = 16) and focus groups (*n* = 11) with a total of 43 managers and staff working in different areas in nursing homes in Austria, the Netherlands and Sweden. The interviews were analysing using thematic coding.

**Results:**

Analysis of the participants statements identified three central elements pertinent to promotion of employee participation and health in the investigated nursing homes. (1) A philosophy and core values of health- and participation-oriented cooperation between managers and staff. This is salient in (2) the orientation on employees’ personal and professional concerns, resources and problems – in particular by promoting skills and trustworthy handling of employees’ work situations, personal issues and sickness – and (3) in the orientation on discussions, empowerment and decision-making in teams mediated through promotion of cooperation and assumption of responsibility within and between teams and support for teams, including in challenging psycho-social situations.

**Conclusions:**

Health- and participation-oriented leadership in nursing homes is an ongoing process that demands strong reflective abilities and organisational skills of those involved.

## Introduction

Health care systems worldwide face challenges as societies age. One of the most important is a growing shortage of workers in health care provision [1, 2]. It is widely accepted that working environments must be improved in order to address this development. This is particularly true in long-term care [3], where persistent staff shortages, funding problems and the growing complexity of users’ health needs have to be addressed [4]. In the past decade European countries have introduced various policies to address these challenges. Reforms have included adapting nursing education, promoting new skill mixes in care teams, improving access to the labour market for foreign care workers and foreign-trained nurses, and measures to improve working conditions and raise salaries [5, 6]. There is also room for action within long-term care facilities themselves. Ensuring that working conditions are conducive to staff health should be a central concern of leadership. Research shows that leadership influences employees’ job satisfaction and health [7, 8]. This also holds true for the nursing workforce [9] and staff in the long-term care sector [10–12].

Management concepts that promote employee participation in efforts to improve well-being and health are known to be beneficial [7]. “Shared”, “distributed” and “collective” leadership styles are increasingly prevalent in health and long-term care settings [13, 14]. Under these concepts management encourages employees to participate more actively in shaping their own working environment, relationships and processes (for example [15]). That is the essence of the health-oriented participatory approach to work and leadership.

Participation is a core principle of health promotion, and has been promoted in community health [16] and in the field of workplace health [17]. In practice, participation is often understood as informing and consulting those whose involvement is desired. But real participation requires collective action and ultimately leads to changes in the distribution of power and authority [18, 19]. Real participation, involving collective action and decision-making can mobilise resources, empower subjects and improve their health and well-being [20].

Despite the potential benefits of health- and participation-oriented leadership, research on how long-term care leaders can facilitate staff participation is still scarce. In their narrative review of the research on “Appropriate leadership in nursing home care”, Zonneveld et al. [21] found that most of the research concentrated on relationship-oriented leadership styles. Relationship-oriented (or relational) leadership involves “using emotional skills such as listening and empathy, to increase the involvement of employees” (p. 20). As Zonneveld and colleagues underline, appropriate leadership is shaped by a multitude of internal and external factors: from personal traits and team-related aspects (such as staff turnover) to structural and cultural conditions.

This leads us straight to the question of the opportunities and challenges associated with realising health-oriented participatory leadership in nursing homes. Nursing homes are traditionally hierarchically organised and are characterised – to different extents in different countries – by state regulation of their structures and processes (including staffing levels, staff qualifications and roles, documentation requirements, etc.) [22]. The proportion of low-skilled workers is typically high (for example [23]). In such a context, employees and managers could perceive demands for greater employee participation as burdensome [17]. In many cases participation is a skill that has to be learned. Furthermore, the obstacles to a health-oriented participatory leadership culture include irregular working hours (like weekend and night shifts), understaffing, high rates of sickness absence, high staff fluctuation rates, and the use of internal cover staff and agency staff (for example [23]). At the same time, the staff bear a great responsibility for the residents’ health and well-being, especially in a situation where nursing home residents tend to have particularly poor physical and mental health (and often also cognition) and to be highly dependent on assistance in their daily lives.

Finally, psychosocial challenges are the norm for those working with nursing home residents and their relatives, not least in connection with death and loss. In view of the pressures under which they operate, including excessive workloads and lack of resources, this frequently creates ethical challenges [24], including in connection with death and dying. These circumstances can cause them to experience moral distress [25, 26], with negative repercussions for their health and job satisfaction [27]. Health-oriented participatory leadership could counteract this through a care culture that allows care teams to collectively address and resolve the ethical dilemmas that arise [27, 28]. That would benefit both quality of care and quality of work life. There is to date, however, a dearth of research on the possibilities for successfully realising health-oriented participatory leadership under the specific conditions found in nursing homes.

## Methods

### Aim of the study

This study investigates health-oriented participatory leadership in nursing homes, exploring experiences in nursing homes in three European countries – Austria, the Netherlands and Sweden – in order to compare approaches and practical implications in different long-term care systems.

Our research questions are:

- How does management promote workplace health through participation?
- What opportunities and challenges are associated with health-oriented participatory approaches?

The present study is part of the research project “BGM Pflege international”, which investigated examples of Good Practice in workplace health management in nursing homes in Austria, the Netherlands and Sweden.

### Study design: qualitative cross-country study

We employed an exploratory qualitative cross-country study design. To collect experts’ “context knowledge” and “process knowledge” [29: 471] on successful health-oriented participatory leadership we conducted semi-structured interviews and focus groups with managers and employees in various functions in nursing homes in Austria, the Netherlands and Sweden. The interviews and focus groups were analysed from a cross-country perspective using thematic coding developed by Flick [30].

Country selection was guided primarily by the prevalence of best practices in workplace health management within national long-term care systems. A comprehensive literature review identified Austria, the Netherlands, and Sweden as particularly suitable cases. The substantial variation in structural characteristics across these systems allowed the analysis to consider the role of such features in the implementation of best practices. The sample includes a variety of long-term-care systems within different types of welfare states [31] to increase the validity of our findings on a broad empirical basis. However, the analysis does not primarily aim to provide a systematic comparative assessment of the effects of institutional care system characteristics on workplace health management in nursing homes.

### Research setting: nursing homes and workplace health promotion in Austria, the Netherlands, and Sweden

Each of the three countries included in the investigation possesses a developed system of professional long-term care and extensive regulation of statutory health and safety at work – which creates openings for voluntary workplace health management [32]. In all three countries workplace health management efforts include primary prevention and health promotion. All three also face similar challenges in securing adequate current and future provision of long-term residential care, in particular the growing proportion of nursing home residents with extensive and complex care needs, persistent skilled labour shortages and rising costs [32]. Lückenbach et al. [32] provide a comparative overview of the organisation and situation of (a) long-term care and (b) workplace health management in nursing homes in these countries. In the following we summarise the essentials.

In Austria long-term care is funded through taxation and private co-payments; any shortfall is covered by the social security net [33]. Responsibility for organising and regulating the long-term care sector lies with the federal states. Nursing homes are run by for-profit, non-profit and public providers. Workplace health promotion is guided by the principles of “health in all policies” [34: 205]. The health insurers provide a uniform nationwide advice service on workplace health management. Employers including nursing homes can have their workplace health promotion programme certified by the Austrian Network for Workplace Health Promotion (“Österreichisches Netzwerk für Betriebliche Gesundheitsförderung – ÖNBGF”) [35].

In the Netherlands long-term care is financed through statutory long-term care insurance and private co-payments. As in Austria, the state backstops the system financially, making up any shortfall not covered by insurance and co-payments [36]. Access to long-term residential care is assessed by the Centre for Healthcare Indication (CIZ), while regional care administration offices (“Zorgkantoren”) commission and oversee private service providers [37]. Commercial provision of long-term residential care is largely excluded in the Netherlands. Since 2017 employers have been legally obliged to retain an occupational health and safety service (“Arbodienst”). However, there is no obligation to conduct workplace health promotion, apart from prevention of accidents and occupational illnesses (which can be bought in from the “Arbodienst” or other providers). Given that employees are able to call off sick without a doctor’s note and sick pay of at least 70% of the salary is paid for up to 104 weeks [38], there are strong incentives for employers to invest in prevention and health promotion for their staff.

The Swedish long-term care system is funded largely through taxation and organised by the municipalities, which are also responsible for planning their local care infrastructure [39, 40]. Each municipality decides whether to provide services itself or buy them in. About 80 % of nursing homes are publicly owned, 18% are commercial and 2 % are run by private non-profit providers [41]. Sweden has had a comprehensive national public health policy since the early 2000s, with the government laying out its work environment strategy every five years [42]. Workplace health and safety management are integrated under the concept of the working environment (“Arbetsmiljo”). Employers are required to conduct systematic management of the working environment [43].

### Sampling and field approach

In order to identify nursing homes that could serve as examples of good practice we conducted semi-structured interviews (*n* = 16) with key informants in the field of long-term care and workplace health management in the three countries of interest [32]. We asked them to name nursing homes that had introduced innovative approaches and/or apparently successful workplace health management. We also searched online for nursing homes of interest and consulted further experts using the snowball method. In Austria, where we lacked sufficient recommendations, we also wrote to all nursing homes whose workplace health promotion programme had been repeatedly certified by the Austrian Network for Workplace Health Promotion and recruited individual homes from this pool. When selecting nursing homes we intentionally included a mix of rural and urban settings and multiple regions.

In order to gain as complete a picture as possible we sought to recruit managers from different tiers (provider organisations, nursing home management, care group managers) and employees with different functions, qualifications and professions. While the details of job titles, profiles and qualifications differ between the three countries, we endeavoured to include skilled workers with academic qualifications or several years of training as well as assistants and auxiliaries with less training or none at all. We contacted nursing home management at all the identified good-practice nursing homes by e-mail and where possible also by telephone. They received written information about the goals and structure of the study and about use and protection of the data. After a preparatory discussion the participants for the study were selected by the nursing home management.

### Data collection: semi-structured interviews and focus groups

We conducted *n* = 16 semi-structured interviews with 24 persons and *n* = 11 focus groups with 43 persons. Altogether 67 subjects participated in the study. Six of the interviews were conducted by a single researcher; ten were conducted by two researchers. The focus groups had three or four participants. The data was gathered between February and November 2023. The researchers visited the nursing homes in person: five in Austria, five in the Netherlands and four in Sweden. In Sweden three further interviews were conducted without visiting the nursing home. Where possible the interviews and focus groups were conducted by at least two of the authors; in ten cases only one member of the research team could be present. All the interviewers were highly experienced in qualitative research and interviewing. The interview guide included the following themes: approaches to and challenges for wellbeing and health of nursing home employees; measures of workplace health promotion/management; strengths and weaknesses. The guide was revised after the first interviews and focus groups, to include specific new questions that arose. The interviews lasted between 43 and 89 min, the focus groups between 26 and 99 min.

All interviews and focus groups in Austria were conducted in German, the native language of the researchers. Those in the Netherlands and Sweden were conducted in English wherever possible. The possibility to conduct interviews in English was clarified in advance with the nursing home management and immediately before the interview with the participants. As well as permitting researchers who do not speak Dutch or Swedish to conduct the interviews themselves, research budget constraints were also involved. Two interviews were conducted entirely in Dutch, in which one member of the research team is fluent. An external interviewer was engaged to conduct two focus groups in Swedish. In the case of the Netherlands, the existence of Dutch language skills in the research team and knowledge of German on the part of a number of study participants allowed misunderstandings to be clarified. That was not possible in Sweden.

The study participants included members of organisation management, nursing home management, head of nurses, care group managers, nurses, nurse assistants and care auxiliaries, social workers, as well as administrators, janitors and cleaners. As far as possible, non-management participants were interviewed separately from their superiors, in order to encourage openness and reduce the risk of social desirability bias in their responses. On account of organisational considerations this was not always possible. Table 1 provides an overview of the participants’ characteristics.

**Table 1:** Participants’ characteristics

	Austria			Netherlands			Sweden		
	female	male	total	female	male	total	female	male	total
**Managers ** **total**	16	1	17	8	2	10	7	1	8
Organisation management	3	1	4	2	2	4	2	0	2
Nursing home management	7	0	7	6	0	6	4	0	4
Care group managers	6	0	6	0	0	0	1	1	2
**Employees ** **total**	7	5	12	5	2	7	13	0	13
Nurses	0	1	1	3	1	4	2	0	2
Nurse assistants	2	1	3	1	0	1	10	0	10
Care auxiliaries	1	0	1	0	0	0	1	0	1
Other health and social professions	1	2	3	1	0	1	0	0	0
Other professions	3	1	4	0	1	1	0	0	0
**Total**	**23**	**6**	**29**	**13**	**4**	**17**	**20**	**1**	**21**

### Ethical considerations

The participants were informed verbally and in writing about the study and the arrangements for recording, storing and processing the data. They were given sufficient time to read and consider the information, and an opportunity to ask questions. They were informed that they were entitled to terminate their participation at any point without stating reasons and to request the deletion of all personal data. Each participant signed the consent statement before recording began. The study received ethical approval from the University of Bielefeld (EUB-2022-201).

### Data analysis: thematic coding

The interviews and focus groups were audio recorded, transcribed verbatim and anonymised. The transcripts of interviews conducted in Dutch and Swedish were machine-translated into English using DeepL^®^ and subsequently checked and edited by a person with very good command of both languages and knowledge in the field of interest. The transcripts were analysed following thematic coding [30] using MAXQDA 2022 (VERBI) software. Thematic coding according to Flick [30; ex. 44] combines an iterative process of open coding [45] of individual cases (transcripts) to build a single-case thematic structure and then made cross-case comparisons to examine similarities and differences between cases – resulting in an overarching thematic structure across cases (i.e. category system). The thematic structure is then applied by coding the whole data material; finally, a fine analysis of the themes comprising detailed interpretation of text passages takes place [45].

In detail, the following steps were applied in this study:

In the first step, a selection of transcripts from all three countries were open-coded and the codes grouped to create themes. This created an initial thematic structure consented in the team (CL, KH, MH, SK). This structure was then applied to the other transcripts and adapted several times in the process (CL, MH).

After this procedure, the coded material was read for further analysis (KH, SK, CL). During this step, the connection of leadership, participation of employees, and employee health crystallised as topic, which could be dimensionalised using the initial thematic structure. The final, consented thematic structure comprises three themes:

1. Philosophy and core values of health-oriented participatory leadership
2. Orientation on individual employees
3. Orientation on teams

All steps so far were performed using MAXQDA. Using a coding book, several coding meetings in the research team took place across all steps to discuss and find a common understanding about the coding process, and to discuss the assignment of text passages in the coding framework until consent was reached.

For the last step, fine interpretation, text passages from each theme were grouped and interpreted (KH, SK) using a text editor programme and final checks and re-assignments of codes were made. For each theme a draft interpretation was distributed to the author team to seek consent via comments and further adaption. The three themes were comprehensively delineated, confirming data saturation.

### Rigour and reflexivity

The research team was composed of three professors, one post-doc and one PhD student (two women and three men), with expertise in nursing science, social sciences, political science and public health. All five have comprehensive experience and skills in qualitative cross-country health research. So we were able to draw on broad and robust methodological and disciplinary expertise throughout the research process. Interview analysis was carried out by four of the researchers in close collaboration. This enabled us to discuss the data in depth until consent on the final thematic structure was reached. Structured reflection throughout the analysis process, including double checking and adaptation of the coding, enhanced the transparency and intersubjectivity of the findings. Because this was a German research team gathering and analysing data in other European countries, linguistic and cultural differences had to be taken into consideration. Before starting to gather data, the research team studied the respective health and long-term care systems in order to properly contextualise the research and findings. This included interviews with experts with deep knowledge of the long-term care and workplace health management systems in the respective countries. We made a point of reflecting on our own position as German academics and our knowledge of the German health and long-term care systems, and maintaining openness towards different practices and arrangements in the countries of interest. Ongoing discussion and exchange in the team ensured that these challenges were addressed consciously. Interviewees were invited to attend an online presentation for member checking of our key findings, but unfortunately this had to be omitted due to a lack of participation.

## Findings

Analysis of the interview material revealed three themes running through the field of participation and health. First of all, there is (1) a basic philosophy of health-oriented participatory relationships between managers and employees. This is salient in (2) an orientation on individual employees (resources, interests, problems) and (3) an orientation on teams (discussions, empowerment, decision-making processes). In the following we outline the relevant aspects of each of these elements and explore the opportunities and challenges associated with them.

### Philosophy and core values of health-oriented participatory leadership

Our interviewees – managers and employees alike – characterised nursing care as intimately bound up with an orientation on health and well-being in all interactions in the nursing home. So health-oriented work and leadership is one element of an overarching care philosophy that encompasses the well-being of care givers and receivers alike. Fundamentally that means

[…] creating a sense of security. But that doesn’t just mean that only the residents should feel safe and secure. Everyone in here should. The residents in the first place, because we are there for the residents aren’t we, but also the staff and ultimately also the relatives. As far as the staff are concerned, if I feel at home and comfortable then I am happy and I can provide good care. And if that’s not the case then everyone notices it, sure. (ÖR4-Int2_P1:65, nursing home management and head of nurses)^1^

Staff need opportunities to communicate their needs. Employees appreciate managers who are open to hearing their problems and suggestions: “[…] keeping the door open and also allowing you to engage in conversation about basically anything you want to say” (NL1-Int2_P3:41, nurse assistant). Managers feel it is important to have a flat organisational structure: to seek dialogue in team meetings, tapping the knowledge of all involved rather than simply giving instructions or holding monologues. Holding back is a learning process for managers, however:

I remember the first time I understood what I needed to change as a leader because I was used to sitting in my office looking through the questions that I’m going to bring to the meeting with my staff. I did the same thing [over and over] and I didn’t change anything. I said I wanted to change and [hear] their opinion but at the same time, I was solving all the problems myself on my own and delivered it. (SWE3-Int1-P1:43, nursing home management)

Employees appreciate when “their” managers take time for them, often without having to be asked. Managers who have an ear for the mood among the workforce and make an effort to speak with staff are regarded as supportive and respectful. This enables them to respond appropriately to situations where employees feel stressed and overworked:

‘How are you getting on? What can I do for you?’. And simply having mutual trust and getting respect. And straight away the work feels easier. And if you say maybe today I’m not feeling so good, then maybe you get given slightly different tasks […] and you pay it back the next day and so on. […] And it’s not just like ‘do it this way, do it that way’ and that’s the end of it. The same way we look at care as a personal, individual thing, they treat us as individuals. (ÖR5-FG4_P3:39, nurse assistant)

Nursing homes in the Netherlands employ academically qualified “quality nurses” (“kwaliteitsverpleegkundigen”) who fill an intermediate role between management and care team. Their functions include promoting quality of care by observing and advising the care teams and supporting the introduction of innovative processes and technologies. The Dutch quality nurses work closely with both carers and management. They sometimes also make use of this intermediary function to communicate employees’ concerns to management. If they are to prevent and resolve conflicts, it is important that they gain the confidence of both sides.

Study participants frequently mentioned conflicts between management and staff. Examples include employees’ criticisms of overly rigid shift plans that are set too long in advance and ignore personal needs, while managers said that understaffing and high rates of sickness absence made their job hard and placed a heavy burden on the remaining staff who had to cover at short notice. One manager complained that employees had made little use of health promotion offers despite intense efforts to ensure they were tailored to their needs. One nurse assistant who was active in the trade union also pointed to the (sometimes) diverging opinions and interests of employers and employees.

Yes, I have to feel good and I’m still healthy and the work should not make me sick. The thing is that the employer has its [own] way for how they see a good working environment. We as workers have another point of view. We have different points of views. Sometimes, we can collaborate and we reach agreement and we can go there. Unfortunately, too many times, we were crashing [failing]. (SWE1-FG1_P1:138, nurse assistant)

Study participants felt that it was crucial to ensure continuous open communication despite (or precisely because of) the conflicts and challenges that inevitably arise in the busy routine. This includes establishing transparent work processes and organisational goals and dealing actively with management decisions.

I think that you can only become a team when there’s full transparency and when you are able to talk about the hard issues with one another. That means people who work together should communicate, but also with the layer above and below. […] There shouldn’t be any secrets in my eyes. That means that you align your goals, […] you can work as a team […]. (NL4-Int1-P1-MG, Pos. 67, management of a group of three nursing homes)

### Orientation on individual employees

Managers feel it is important to treat their staff as individuals and to seek regular contact. They try to take a holistic perspective on the employee’s working situation, including their sensemaking at work and quality of life as a whole. In addition to providing opportunities to communicate in the everyday routine, management at almost all the investigated nursing homes conducted annual staff appraisals to discuss job satisfaction and explore any need for change. In doing so, they hoped to enable employees to make the most of their abilities and avoid both overwork and underemployment.

I speak with the staff about how much they still enjoy their work. But I also try to give people tasks they will enjoy. One person might like preparing shift plans, while another prefers to be dressing wounds. (NL3-Int1_P1:103, nursing home management)

Many managers are keen to ensure their staff have opportunities for training and promotion, in order to improve their skills and enhance their job satisfaction – and by so doing to improve staff retention. In Sweden they can access government programmes for improving qualifications in the care sector that enable participants to combine work and training. This keeps them in their existing jobs while improving their qualifications, to the benefit of both employee and employer. In the longer term this can lead to promotion into management positions.

They [care auxiliaries] are working 50%, getting an education 50%, and getting 100% salary and that’s a perfect way. Next year we have 22 new assistant nurses. (SWE6-Int1_P2:302, nursing home management)

One of the central responsibilities of leadership is to support staff who are experiencing personal difficulties. Ideally this will benefit both the individual and the situation in the workplace. The cause of the problems may lie in the situation at work or in the employee’s personal life. For people working in nursing homes reaching – or exceeding – their physical or mental limits is more or less daily routine, whether through conflicts with individual residents and/or their relatives or specific situations such as dying processes. If staff are to feel satisfied and secure in their work it is crucial that they are able to access timely expert support in such situations, and that they are not left to cope with stress alone. One nurse assistant in Austria commented:

But my cry for help created a situation where we are able to speak openly and honestly about these problems. We brought in experts and everyone in the team – whether young or old, new or already there for longer – said what they think about this. And in the end, in my opinion, we did find a good solution for the final phase of their life. So I was then going home happier with the quality of my care. And that improved my mental health, because I was able to switch off properly and I wasn’t fretting about it all any more. At the time it had really been getting to me. So I had been looking in books and in my notes from college and researching whether there was anything I could do. But we don’t want that. You have to be able to switch off too. (ÖR5-FG4_P3:98, nurse assistant)

Managers regard offering support on relevant issues – including aggression and violence – as part of their duty of care towards employees.

Well even here 67 % of our residents currently have a dementia diagnosis, and many of those are geronto-psychiatric forms of dementia. And that means that we need […] specialised staff. Now that requires a lot of investment in training and coaching […], because otherwise the staff just won’t be able to cope. Especially if we are also thinking about the question of violence, when we are looking at these problematic forms of dementia. That certainly demands a lot of time from the staff, and energy too. (ÖR4-Int2_P1:11, nursing home management and head of nurses)

It is not uncommon for staff to have private problems. These may be family-related, financial or psychological. The study participants observed that the latter generally tended to affect younger members of staff. Because these and other private problems often have a direct impact on personal performance, job satisfaction and the work atmosphere as a whole, managers often work with the affected person to come up with solutions. Here the manager’s ability to communicate and reflect is crucial.

Well if I see behaviour changing, for example if someone is suddenly working a lot more hours. Then I ask myself why someone might suddenly start working so much. And then later I received a garnishment order, meaning that they had debts to pay. So then I spoke with them about it and then we also try to help them with their debts, because otherwise it will only cause trouble. (NL3-Int1_P1:55, nursing home management)

The interviews revealed a great desire to improve employees’ work/life balance. The central issue in nursing homes is flexibility and personalisation of shift scheduling. In recent years, some of the investigated nursing homes had adopted new flexible strategies to improve employees’ work/life balance, reduce stress and thus improve staff retention. These include personalised shift planning and employment contracts with different numbers of hours.

Sick leave is high on the agenda for managers and staff alike. The principal issues are preventing absences, dealing with those who are off sick, and finding strategies for reintegration after long-term sick leave. First of all many managers see it as their responsibility to avoid and prevent stress and overwork (in particular but not only for older staff), with positive effects on sickness absences.

But as a manager I believe that the most important thing is to start the discussion before it’s too late. […] I am also a vitality coach, so I have nobody who is over 60 absent because they can’t keep up physically. Before it gets that far I will have spoken with them to focus their thoughts. Sometimes people get angry when I say they can’t go on as they are. But during the past year I have managed to get four people into a different roles that way. […] One of them suddenly had trouble with her shoulder. She’s now a hostess so she no longer has to do hands-on patient care. Now she is only responsible for tea, coffee and activities. […] That way I avoid absences, and I also avoid her becoming dissatisfied with her work. (NL3-Int1_P1: 59–61, nursing home management)

The overtime associated with high rates of sickness absence is stressful for managers and employees. But both sides agree that sickness should be handled confidentially. In the Netherlands in particular, being contacted by a manager when off sick was regarded positively.

I think there is also a trust from [nursing home management] when someone calls in sick, that that person is really sick. I think that if that person were really sick, they would find it very annoying not to hear from the supervisor for a fortnight ‘How is it going?’ (NL3-Int2_P2:72, quality nurse)

Longer absences are associated with additional challenges. Managers emphasised the necessity for sensitive and flexible arrangements for staff returning to work. It was regarded as helpful to return to work in some form as early as possible in order to stay in touch. The point here is to arrange the return to work in such a way as to ease the employee back in and allow them to increase their hours gradually, for example starting with 25 %. But that demands coordination on the management side and cooperation by colleagues – and can be a source of stress in situations of staff shortages.

Over and above finding viable solutions for staff returning from (long term) sick leave, the general level of sickness absences is an issue for team leaders and management across all three countries. We found different cultures and routines in connection with sick leave. Some managers described employees’ attitudes as lackadaisical.

P1 (nursing home management): It isn’t just the manager’s responsibility. They are a team. Maybe someone comes in the morning and she isn’t well and she has made a big effort to come to work. And then you say ‘Better go home again you aren’t well’ or ‘We’ll help you get through the day’.

P2 (director, management of a group of nursing homes): If someone says ‘I’ve got a headache’ their colleagues always tend to say ‘Oh you’re ill, you’d better go home’. And then we say ‘No, just take a Paracetamol and wait for a moment, maybe half an hour or so, and then you’ll be able to get on with your work’. (NL2-FG1_P1-P2:248)

Where managers show the latter attitude staff may feel fearful of calling in sick. This can lead to presentism and poor outcomes for the sick individual, for their colleagues, for the nursing home residents and for the nursing home as a whole.

Other managers emphasised that their responsibility for the organisational culture was decisive for job satisfaction and identification – and thus ultimately for the level of sickness absences.

You have to have the courage when you are a leader to talk about what kind of culture we have here in [unit I]. Sometimes it could be […], if you don’t feel like you are meaningful in your work, if you don’t feel like you’re proud, it will be really easy not to go to work. If you feel like you have a bit of a headache, but you’re proud – that matters a lot. (SWE6-Int1_p2:298, nursing home management)

### Orientation on teams

The tasks involved in caring for residents are always a matter of teamwork. So the question of working in and with care teams was a major issue in the interviews and focus groups. According to managers and employees alike it makes a big difference for job satisfaction and health if team meetings are focussed and relevant: “Little things that shift the discussion culture a little bit (…), have had much more effect than […] something like the yoga course.” (ÖR6-FG6_P4:115, nursing home management).

The composition of teams changes frequently, not least because of staff fluctuation. One nursing home in Austria held teambuilding days where groups with many new members participated in organised activities outside the workplace. Managers in Austria and the Netherlands also reported that they had recently moved to conducting joint shift handovers across multiple residential groups so that each team learned about the others’ situation and challenges, and ideally also supported them.

That’s something we introduced last year because […] there was no communication between the teams. When one team had two people sick and someone was all alone, and another team had someone extra, they didn’t know about it […]. We [the management] learned about it the next day then because someone was really upset and the others were really relaxed. But we try to address it beforehand now. (NL4-Int1-P1:94, management of a group of three nursing homes)

While managers certainly feel it is important to be available to handle the unavoidable conflicts that arise they would ideally like their teams to be able to resolve problems and tensions on their own.

Naturally also the mental health, that we don’t forget that. Also for the staff, specifically in case conferences. For example, […] if we have residents with challenging behaviour, we need to discuss the case together and think about how we can make it easier for all involved (ÖR1-FG1_P2:21, care group manager)

The interviews and focus groups reveal a belief that the staff teams are often able to come up with better solutions than a manager would be able to as an “outsider”. On the other hand there is also a risk of dysfunctional forms of interaction becoming established and disrupting the work of the group, for example if individual members dominate the discussion (or conversely feel excluded). In Dutch nursing homes the aforementioned quality nurses intervene where groups are unable to resolve issues satisfactorily on their own.

The coordinating nurse, she has the overview of that so she can raise the alarm, when things are really not going well there, if necessary. But as a team you are also responsible yourself. Just stay open and honest with each other. (NL1-Int2_P3:114, nurse assistant)

Study participants found external supervision and coaching helpful if “there is trouble brewing in a team or you can sense that something is up […]” (ÖR1-FG1_P2:21, care group manager). If teams with problems are actually to make use of external support, it is very helpful if they are already familiar with it and know from experience that it is helpful. One nursing home manager in the Netherlands reported how it took some time for the work with external coaches to become established.

P2: In the beginning, there was a lot of resistance, but we do it for quite a few years now. This year this was our discussion. The form has a bit changed, but it’s part of the game now. Everybody is used to it, and it took a few years. […]

Interviewer 1: They get used to it, but that means also they use it?

P2: Yes, they do. […] Sometimes you see when people are a bit changed in the team. Working together is needed. Sometimes they say, can we get the coach because we have to make new arrangements together and we find it difficult, so they ask, can we get a bit of support? (NL1-Int1_P2:108–113, nursing home management)

In general managers in the investigated nursing homes felt that it was helpful for employees’ health and well-being if teams were able to make their own decisions about how to organise their work. This is associated with an expectation that staff will do more than simply carry out tasks assigned to them. Instead staff are expected to assume responsibility within their teams and contribute to the success of the organisation. As some of the managers reported, this is certainly a learning process. They underline that staff need to be able to experiment and learn from mistakes, and that they need to be supported by their own managers, as well as offers of training and/or coaching. They point out that staff competence is often underestimated in the long-term care sector and that more can often be expected of staff with low qualifications or none at all.

Interviewer: You were discussing the spending of the budget with the staff?

P1: Right. I also gave them budget. Every group got their spending budget for the materials that they use. Food and also a few other things they need to buy, to organise […] We have to start to change the way we look at their [the nurse assistants’] input and their responsibility because they want to take responsibility. […] They know how to do this. Budget is nothing they don’t understand, they do understand it if I give them the tools. I said we work much better together and it’s much easier to hold the budget because then the staff start to think as a manager. (SWE3-Int1_P1:51–58, nursing home management)

If staff are able to cope with more demanding roles, having greater responsibility contributes to greater job satisfaction.

I love it. My place, [it’s] my second home. […] It was when I started as a nurse. I just did that job only. Now I’m free. I can buy something [for work]. I am more free. (SWE6-FG1_P2:113–115, nurse assistant)

## Discussion

Our study examined how management promotes workplace health through employee participation and explored the opportunities and challenges associated with such approaches. For our study participants, leadership orientated on health and participation is guided by (1) a basic philosophy and core values of appreciation and open transparent communication. On that basis, managers orientate on (2) individual employees and (3) organise and support teams. Employees feel it is crucial to be able to discuss the issues that matter to them freely with their managers. They need to know that their problems and needs are taken seriously and that their work is appreciated. If that is the case they are able to cope with the challenges of work and collectively generate a health-promoting work environment.

In our interviews and focus groups we encountered numerous examples where managers encouraged employees to contribute their own perspectives and opinions, to participate actively in shaping their work environment and processes, and to speak openly about work-related and personal challenges and problems. This gave employees a sense of appreciation and confidence that was associated with lower levels of stress, stronger motivation and opportunities to reflect on their own health at work. As such our findings align with prior research on the positive relationship between participatory leadership styles and employees’ health and motivation [13, 14].

As our findings show, the basic philosophy and core values of appreciation and openness communicated by managers is central to employee participation. In the first place, promoting participation through leadership means working on the relationship between the manager and their employees and teams. But collegiality and work on relationships is also required within and between teams. Respondents connected their orientation on relationships explicitly with the nature of care, which relies on trust, empathy and appreciation [46] and forms the basis for fulfilment at work. Experiencing one’s work as worthwhile and meaningful, providing best care and experiencing success [47] are central aspects of “healthy work” [48, 49].

The elements of health-oriented participatory leadership identified in our study include elements of relationship-oriented, supportive and transformational leadership styles. These are, according to the systematic review by Niinihuhta & Häggman-Laitila [50], positively associated with nurses‘ work-related well-being. Likewise, the systematic review by Cummings et al. [9] reported “robust findings to support that relationship focused leadership practices are linked to better outcomes for nurses related to their work environments, their perception of and performance in their workplace, and importantly, their personal health and well-being” (p. 50). However, Zonneveld et al. [21] note that these positive effects are not found by all studies on relationship-oriented leadership.

According to the findings of our study, managers need strong personal reflection skills to meaningfully promote workplace health through participation. Many of the managers we interviewed reflect on their own leadership styles and their effects on employees, and are motivated to improve their skills. They also expressed great willingness to genuinely involve employees in decision-making. Employee participation functions through formal and informal channels of communication and participation at individual and team level (up to and including possibilities for shaping their work and operating independently and autonomously). In terms of the categories laid out by Wright [18], the interviewees’ reports lie in the range “involvement” – real “participation” – “self-organisation”. At the same time a foundation of trust and appreciation strengthens the ability of teams to address tensions and conflicts openly and search for solutions collectively. Similar, Tyler & Parker [51] showed that simply mandating teamwork is ineffective; instead it needs to be backed up, confirmed and consolidated by the attitudes and behaviour of the managers.

As Zonneveld et al. [21] point out, leadership in nursing homes is a multidimensional affair influenced by a multitude of internal and external factors. Leadership is influenced by the manager themself, the teams and the organisation. Our findings indicate that a high degree of participation (or even self-organisation) of employees without appropriate support by management can be counterproductive. This needs to be taken into consideration, especially in view of the high proportion of assistants and auxiliaries in the long-term care sector. The often significant proportion of non-native-speakers can also hinder the development of relationships and restrict the possibilities for participation. Our findings indicate that both managers and employees believe that it is important for healthy working environments that management remains in close touch with the practical work. This is also echoed in the literature where remoteness from practice [52, 53] and the existence of multiple tiers of management [54, 55] are identified as barriers to employees’ participation and practice change.

Health-oriented participatory leadership is always work in progress. Once it has been established it must still be tended and maintained. Given the prevalence of part-time working and unpopular working hours – such as night and weekend shifts – managers in nursing homes need to ensure that they maintain viable contact with all staff, which may require additional effort to initiate and maintain relationships. High rates of fluctuation are also disruptive for team-building [56], while – according to our participants – health-oriented participatory leadership improves job satisfaction and thus also helps to reduce fluctuation. Managers and employees require strong social skills and empathy [57] and a broad repertoire of formal and informal instruments for maintaining contacts and generating opportunities for participation [58]. Participation-oriented leadership presupposes strong management skills and qualifications (including the aforementioned reflection skills). This is particularly important in connection with appropriate handling of short-term and long-term health-related absences. As the interviewed managers and employees confirmed, dealing with high rates of sickness absence in a situation of staffing shortages and recruitment difficulties represents a challenge and often a source of conflict.

Altogether our study identifies more similarities than differences in the understanding of health-oriented participatory leadership in the investigated good practice nursing homes in Austria, the Netherlands and Sweden. However, there are differences in the importance placed on particular elements, which point to specific differences between the countries:

In Austria the importance of philosophy and core values was mentioned frequently in the interviews and discussions, emphasising its importance for quality of care and staff wellbeing. In Sweden participants emphasised the question of transferring decision-making to staff and the role of co-determination in workplace health promotion and beyond. In the Netherlands participants devoted more attention to the question of absences for sickness, where they reflected on the role of management and addressed the importance of balancing empowerment and responsibility for employees’ health and wellbeing at work.

### Limitations

The study is a good practice analysis, where the investigated nursing homes were selected on the basis of expert recommendations (and in the case of Austria a preselection on the basis of external certification). We are not able to say anything about the scope and dissemination of the identified practices. In terms of the interview methodology we oriented ourselves towards Lincoln and Guba’s [59] four pillars of trustworthiness. Therefore, we implemented data and researcher triangulation (credibility), closely analysed and described the context of our study (transferability), comprehensively documented our data collection process and functions of our interview partners (dependability) and reflected our results within our interdisciplinary research team and other experts (confirmability). However, some challenges remain: (1) Interview language and translation: most of the interviews and focus groups in Sweden and in the Netherlands were conducted in English. In the case of the Swedish data, some of the statements were less clear and precise than we would have wished, presenting challenges for interpretation and analysis. (2) Interviewers’ background: different members of our multidisciplinary team conducted the interviews, including one external interviewer who was carefully briefed regarding the content and concept of interviews and focus groups. Most of the times two interviewers were present to prevent one-sidedness so that the risk of interviewer differences was addressed. (3) Participants professional background: We intended to include participants with different job profiles and levels of qualification. However, in Sweden and the Netherlands (with a single exception) all the participants worked in care and nursing, with none from other areas like social work or housekeeping. Altogether we were able to recruit very few low skilled and unskilled employees.

For future studies we would recommend targeted recruitment of underrepresented groups for one-on-one interviews and/or homogenous focus groups. This would require interviewers fluent in the participants’ native languages. Participant observation would also offer promising possibilities for supplementing verbal data.

## Conclusions

Successful health-oriented participatory leadership in nursing homes requires managers who are sensitised, trained and qualified. Their role is to communicate an open and appreciative stance and lead by example in order to ensure that the approach is understood, accepted and adopted by their staff. If employees are to assume greater responsibility for organising their work, the process must be developed collectively. This demands strong reflective abilities and organizational skills by all persons involved and should be backed up with proper training. As this study demonstrates, health-oriented participatory leadership, when implemented consistently, contributes to greater motivation and lower absence rates, and improved staff retention.

## Data Availability

The data that support the findings of this study are available on reasonable request from the corresponding author. The data are not publicly available due to privacy and ethical restrictions.

## Footnotes

1 Direct quotes from interviews have been linguistically edited to improve readability.
